# ZO-1 and ZO-2 Are Required for Extra-Embryonic Endoderm Integrity, Primitive Ectoderm Survival and Normal Cavitation in Embryoid Bodies Derived from Mouse Embryonic Stem Cells

**DOI:** 10.1371/journal.pone.0099532

**Published:** 2014-06-06

**Authors:** Dominic C. Y. Phua, Jianliang Xu, Safiah Mohamed Ali, Adrian Boey, Natalia V. Gounko, Walter Hunziker

**Affiliations:** 1 Epithelial Cell Biology Laboratory, Institute of Molecular and Cell Biology (IMCB), Agency for Science Technology and Research (A*STAR), Singapore, Singapore; 2 IMB-IMCB Joint Electron Microscopy Suite, Agency for Science Technology and Research (A*STAR), Singapore, Singapore; 3 Department of Physiology, National University of Singapore and Singapore Eye Research Institute (SERI), Singapore, Singapore; Emory University School of Medicine, United States of America

## Abstract

The Zonula Occludens proteins ZO-1 and ZO-2 are cell-cell junction-associated adaptor proteins that are essential for the structural and regulatory functions of tight junctions in epithelial cells and their absence leads to early embryonic lethality in mouse models. Here, we use the embryoid body, an *in vitro* peri-implantation mouse embryogenesis model, to elucidate and dissect the roles ZO-1 and ZO-2 play in epithelial morphogenesis and *de novo* tight junction assembly. Through the generation of individual or combined ZO-1 and ZO-2 null embryoid bodies, we show that their dual deletion prevents tight junction formation, resulting in the disorganization and compromised barrier function of embryoid body epithelial layers. The disorganization is associated with poor microvilli development, fragmented basement membrane deposition and impaired cavity formation, all of which are key epithelial tissue morphogenetic processes. Expression of Podocalyxin, which positively regulates the formation of microvilli and the apical membrane, is repressed in embryoid bodies lacking both ZO-1 and ZO-2 and this correlates with an aberrant submembranous localization of Ezrin. The null embryoid bodies thus give an insight into how the two ZO proteins influence early mouse embryogenesis and possible mechanisms underlying the embryonic lethal phenotype.

## Introduction

The epithelial tissue is one of the main types of tissue in the human body. It lines the external body and organ surfaces, providing a permeability barrier that protects against the external environment. The internal cavities of organ systems are similarly lined and compartmentalized into functionally distinct partitions through the selective regulation of ionic and molecular exchange between luminal and interstitial compartments, thus creating separated tissue microenvironments.

Central to this permeability barrier function is the organization of individual epithelial cells into an epithelial sheet (the epithelium) by cell-cell junctions that regulate paracellular movement and the coordinated apico-basal polarization of this sheet into functionally discrete subcellular regions, which facilitate vectorial transcellular transport. A hallmark of epithelial cell-cell junctions is the tight junction (TJ). This structure forms a network of anastomosing intramembranous strands encircling the apico-lateral domain of the epithelial cell, eliminating the paracellular space between adjacent cells. This tight lateral seal is thus responsible for the epithelial paracellular permeability function [1].

The gatekeepers of this charge- and size-selective permeability function are the TJ integral transmembrane proteins which both cis-multimerize intramembranously and engage in extracellular trans-interactions with their adjacent-cell counterparts. Key TJ transmembrane proteins are members of the Claudin family [2], Occludin [3], Tricellulin [4] and MarvelD3 [5]. Of these proteins, the Claudin protein family members are necessary and sufficient for both the TJ structural strand formation and the selective paracellular permeability function [6].

The trans-association of TJ transmembrane proteins across adjacent cells is stabilized by the simultaneously association of their intracellular domain with submembranous scaffold proteins. The latter proteins in turn bind the underlying actomyosin cytoskeleton, thus mechanically anchoring the TJ complex. These peripheral scaffold proteins are also multi-modular adaptors that interact with various structural and regulatory proteins, forming signaling platforms involved in diverse signal transduction pathways [7].

Functionally important TJ scaffold proteins are the Zonula Occludens (ZO) family of proteins, consisting of ZO-1 [8], ZO-2 [9] and ZO-3 [10]. These three multi-modular proteins belong to the membrane-associated guanylate kinase-like (MAGUK) family and are structurally characterized by three N-terminal PSD-95/discs-large/ZO-1 (PDZ) domains; the central Src homology 3 (SH3) and guanylate kinase-like (GUK) domains; and a proline-rich domain [11]. Crucial to strand assembly, these protein-protein interaction domains confer a structural role by associating with the cytosolic tails of TJ transmembrane proteins and F-actin. Aside from such passive scaffolding functions, ZO proteins have regulatory roles and are known to interact with several cell polarity and actomyosin regulators, signaling proteins and transcription factors [12]. Furthermore, under conditions of low cell confluency or junctional remodeling, some ZO proteins can shuttle between the TJ and nucleus [13]. Therefore, these features allow the ZO proteins to act as mechanosensors of extracellular changes impinging on TJ dynamics by coordinating junctional assembly with basic cellular processes like cell polarization, proliferation and differentiation [14] [15].

Recent studies in cultured epithelial cells have indicated the significance of ZO proteins in epithelial morphogenesis and junctional biology, in particular ZO-1 and ZO-2. The dual suppression by gene-deletion (knockout) and protein-depletion (knockdown) of ZO-1 and ZO-2, respectively, in mouse mammary epithelial cell-line EpH4 was sufficient to abolish the assembly of TJ strands and thus, the permeability barrier function [16]. In this context, exogenous expression of either two proteins rescued the mutant phenotype, thus exhibiting functional redundancy of ZO-1 and ZO-2. Notably, the absence of TJs did not affect apico-basal polarization. In a similar study done on canine kidney epithelial cell-line MDCK [17], although TJ presence was not abolished, protein-depletion of both ZO-1 and ZO-2 led to increased macromolecular solute permeability and abnormal barrier remodeling kinetics. In addition, the organization of the apical circumferential actomyosin ring was compromised. This was associated with an irregular epithelium organization in which cells were laterally misaligned and the apical domain was distended.

The importance of these two ZO proteins is further emphasized in *in vivo* mouse models in which either ZO-1 or ZO-2 gene knockouts resulted in embryonic lethality. ZO-1 gene-deleted mice were embryonic lethal at E10.5 to E11.5. This was associated with a defective organization of the notochord, neural tube and allantois, resulting in extensive apoptosis. Additionally, angiogenesis in the yolk sac was deficient despite normal endothelial cell layer differentiation [18]. Similarly, ZO-2 null mutant mice also exhibited embryonic lethality, but at an earlier stage of developmental arrest from E6.5 compared with ZO-1 null mice. This earlier lethality is possibly due to a compromised TJ structure and barrier function, which in contrast remained unaltered in the ZO-1 null. ZO-2 null lethality was preceded by a reduced proliferation of the embryo at early implantation E6.5 and increased apoptosis at E7.5, leading in failure to gastrulate [19]. Contrary to both ZO-1 and ZO-2 null phenotypes, ZO-3 gene-deletion in mouse and cell-line models revealed no observable abnormalities, indicating a dispensable function of ZO-3 [20] [19].

Generally, the mouse ZO-1 or ZO-2 null models allude to non-redundant independent roles of these proteins in early embryonic development, whereas cell-line models imply a dispensable role of either protein in TJ structure and function. There are however exceptions to this. In MDCK cell-line, sole depletion of ZO-1 protein displayed increased paracellular permeability to non-ionic large solutes [21]. Compromised permeability was also evident after ZO-2 protein-depletion, in addition to abnormal F-actin localization and delayed membrane recruitment of junctional proteins [22]. The differences in interpretation of ZO redundancies and functional roles could reflect experimental variations in ZO protein-depletion levels or context dependence.

Embryonic stem cells (ESCs) are pluripotent and can differentiate into the epithelial cell lineage, among others. ESCs can be cultured to generate embryoid bodies (EB) [23] [24]. EBs are suspension cultured 3D spherical cell clusters of ESCs, which spontaneous differentiate into an outer-layer epithelium (primitive endoderm), a basal extracellular matrix (ECM), and an inner-layer epithelium (primitive ectoderm) surrounding an interior cavity. This morphologically and biochemically recapitulates mouse peri-implantation embryogenesis until the egg cylinder stage [25]. Thus, this makes EBs a useful *in vitro* model to study epithelial morphogenesis and *de novo* TJ assembly in the context of early embryonic development.

To further dissect the distinct roles and redundancies of ZO proteins and also gain insight into the underlying mechanisms responsible for the observed embryonic phenotypes in the ZO-1 and ZO-2 knockout mice, we generated homozygous knockout mouse embryonic stem cells (mESC) deleted of the individual ZO-1 or ZO-2, and both genes in combination. We report the loss of TJ assembly in the epithelium of homozygous ZO-1 and ZO-2 double knockout EBs. This is associated with epithelium disorganization and macromolecular permeability. A discontinuous deposition of ECM by the epithelium results in irregularly formed cavities and accumulating apoptotic bodies. There was no observable phenotypic difference between ZO-2 null mutant EBs and wild-type (WT) control. However ZO-1 EB knockouts displayed mutant phenotypes intermediate between the double mutant and WT.

## Materials and Methods

### Generation of ZO-1 and ZO-2 null mutant mESCs

Mouse W4 ESCs (Taconic) were used in the studies described in this report. The generation of homozygous ZO-1 (ZO-1^-/-^) and ZO-2 (ZO-2^-/-^) null mESCs has been described previously in [26] and [19] respectively. Homozygous combined ZO-1 and ZO-2 null (ZO-1^-/-^ ZO-2^-/-^) mESCs were generated as follows. Two independent ZO-1^-/-^ mESC clones were separately transfected with puromycin-resistant pCre-Pac plasmid [27], which expresses Cre protein that specifically deletes the floxed Neo gene from the ZO-1 targeted alleles. After selection in puromycin for 24 hrs, half of the cells from the surviving clones were cultured in normal ESC medium as a master plate and the other half in 500 µg/ml G418 for negative selection, to confirm Neo gene-deletion. The Neo-negative clones were next transfected with the LacZ-Neo-containing ZO-2 targeting construct to generate double ZO-1^-/-^ ZO-2^+/−^ mESCs, and subsequently selected in a low concentration of 250 µg/ml G418 and screened. To obtain ZO-1^-/-^ ZO-2^-/-^ mESCs, the ZO-1^-/-^ ZO-2^+/−^ mESCs were selected with a high concentration of 10 mg/ml G418 (Calbiochem). Drug-resistant clones were picked after 7-9 days of selection. Half of the cells from each clone were polymerase chain reaction (PCR)-screened and the rest were expanded on feeder cells as a master plate. Southern blot further verified correct targeting in the PCR-screened clones and immunoblot confirmed the absence of ZO-1 and ZO-2 proteins. Two independent mESC clones of ZO-1^-/-^, ZO-2^-/-^ and ZO-1^-/-^ ZO-2^-/-^ knockout were used in the investigations reported here. Wild-type (WT) mESCs served as controls.

### Mouse ESC and EB culture

mESCs were cultured without feeder cells in 0.3% gelatin (Calbiochem) -coated plates with ESC medium consisting of DMEM, high glucose (Gibco), 20% foetal bovine serum (FBS) (HyClone), 100 U/ml penicillin, 100 µg/ml streptomycin, 0.1 mM non-essential amino acid, 1 mM sodium pyruvate (Invitrogen), 0.1 mM β-mercaptoethanol (Sigma-Aldrich) and 1000 U/ml ESGRO Leukaemia inhibitory factor (LIF) (Millipore), and maintained at 37°C in 7.5% CO_2_. EB medium is similar to ESC medium except that LIF was omitted and only 10% FBS was used. EBs were cultured by seeding 1000 mESCs in 20 µl hanging droplets of EB medium in a humidified plate at 37°C in 7.5% CO_2_ for 3 days. Subsequently, the newly formed EB spheroids were transferred into a suspension culture of EB medium for continued incubation, and harvested at specific time points for analysis. EB development was tracked and images were captured digitally using the Eclipse TE2000-S inverted microscope mounted with a DS-5Mc camera (Nikon).

### Antibodies and reagents

Primary antibodies and fluorophore-conjugated or horseradish peroxidase (HRP)-conjugated secondary antibodies applied in immunofluorescence (IF) or immunoblot (IB) analyses are listed in Tables B and C respectively in Figure S1. Nuclei were stained with 4,6-diamidino-2-phenylindole (DAPI) (Invitrogen).

### Southern blot analysis

Genomic DNA was extracted from G418-resistant ESC clones and completely restriction digested with ScaI-HF (New England BioLabs). For the ZO-1 locus, a 554 bp ZO-1 probe, corresponding to the 5′ arm of the targeted allele, was used to detect the 10 kb and 14.8 kb bands from the WT and mutant alleles respectively. For the detection of the respective 6.7 kb and 11.5 kb bands of the WT and mutant ZO-2 alleles, a 566 bp ZO-2 probe corresponding to the 5′ arm of the targeted allele was used. These probes were generated from PCR amplification of WT control genomic DNA using the specific primers listed in Fig. S1. According to the manufacturers' protocols, the probes were labelled using the Digoxigenin (DIG)-DNA Labeling kit (Roche) and hybridized to nylon membrane Hybond-N (GE Healthcare)-Southern transferred ScaI-HF digested genomic DNA. Hybridized probes were visualized using the DIG Luminescent Detection kit (Roche).

### Cell lysis and immunoblot analysis

ESCs or EBs were lysed in a modification of the radio-immunoprecipitation assay (RIPA) lysis buffer (50 mM Tris, pH 7.4, 150 mM NaCl, 1% Triton X-100, 1% Sodium deoxycholate, 0.5% Sodium dodecyl sulphate (SDS)), supplemented with phosphatase inhibitor, PhosSTOP and complete protease inhibitor cocktail (Roche). Lysates were sonicated and centrifuged at 13,200 rpm for 20 min to obtain soluble fractions. These were separated by SDS-polyacrylamide gel electrophoresis (SDS-PAGE) and transferred by Western blot onto nitrocellulose membrane Hybond-C Extra (GE Healthcare). Membranes were then blocked with 5% skimmed milk in 0.1% Tween 20 in phosphate-buffered saline (PBS) and incubated with appropriate primary and HRP-conjugated secondary antibodies in 1% skimmed milk in 0.1% Tween 20 in PBS. Membranes were visualized by chemiluminescence using Super Signal West Pico (Pierce).

### Immunofluorescence analysis

Two fixation methods were used; paraformaldehyde (PFA) or methanol fixation. For PFA fixation, fresh EBs were fixed in 4% PFA for 30 min to 1 hr and sequentially treated with 7.5% sucrose for 3 hr and 15% sucrose overnight. Fixed EBs were then embedded in Tissue-Tek O.C.T. compound (Sakura) and cryosectioned at 5 to 8 um thickness. The sections were quenched with 50 mM ammonium chloride and permeabilized with 0.2% Triton X-100 in PBS. For methanol fixation, fresh EBs were immediately embedded in Tissue-Tek O.C.T. compound (Sakura), cryosectioned at 5 to 8 um thickness and fixed in ice-cold Methanol for 20 min. Both permeabilized-PFA fixed and methanol fixed sections were blocked in 1% bovine serum albumin in 0.1% Triton X-100 in PBS. These were subsequently incubated in primary antibodies and labelled with appropriate fluorophore-conjugated secondary antibodies. Images were acquired using an Axio Imager.D1 upright microscope coupled to an AxioCam MRm digital camera (Carl Zeiss).

### Scanning electron microscopy (SEM) and Transmission electron microscopy (TEM) analysis

For SEM analysis, EBs were fixed in 2.5% Glutaraldehyde in 0.1 M phosphate buffer, pH 7.4. Post-staining was done in 1% Osmium tetraoxide before gradual dehydration in increasing ethanol concentrations. Dehydrated EBs were dried in a critical point dryer before being mounted and sputter coated with gold (Leica). Samples were viewed in a Jeol 6701F field emission SEM. For TEM analysis, EBs were fixed in 2.5% Glutaraldehyde, 4% PFA and 0.2% Picric acid in 0.1 M Sodium cacodylate buffer, pH 7.6. Post-staining in 1% Osmium tetraoxide and 1.5% Potassium ferricyanide diluted in 0.1 M Sodium cacodylate buffer pH 7.6 for 1 h was done before gradual dehydration in increasing ethanol concentrations. Then samples were stained with 1% Uranyl acetate in 100% ethanol, before being embedded in Spurr's resin. Ultrathin sections were counterstained with 4% uranyl acetate and Reynold's lead citrate and examined with a JEM-1010 electron microscope operated at 80 kV or a JEM-2200FS operated at 100 kV.

### Endoderm permeability assay

EBs in fresh EB medium were pre-incubated with rabbit anti-Laminin 1+2 (abcam) or rat anti-Perlecan (Millipore) at 10 µg/ml for 2 hrs at 37°C in 7.5% CO_2_. The EBs were then harvested, washed in PBS, fixed with 4% PFA and sectioned as described in the ‘Immunofluorescence analysis’ protocol. 0.2% Triton X-100 permeabilized sections were subsequently treated with appropriate fluorophore-conjugated secondary antibodies to visualize any pre-incubated antibodies present in the sections.

### RNA extraction, semi-quantitative RT-PCR and quantitative PCR

According to manufactures' protocols, total RNA was isolated from EBs using the RNeasy kit (QIAGEN). Semi-quantitative reverse transcription-PCR (RT-PCR) analysis was carried out with the OneStep RT-PCR kit (QIAGEN). For quantitative real-time PCR (qPCR), cDNA was reverse transcribed from isolated total RNA using the Maxima First Strand cDNA Synthesis kit (Thermo Scientific). qPCR was then carried out with the KAPA SYBR FAST kit (Kapa Biosystems). The specific flanking-primer sequences for each gene and expected amplicon size are listed in Fig. S1.

### Histological analysis

EBs were pre-embedded in 1.5% agarose and subsequently fixed in 4% PFA for 1 hr to overnight, and gradually dehydrated in increasing concentrations of ethanol, with a final xylene treatment before final embedding in paraffin. 10 um sections of embedded EBs were stained with Hematoxylin and Eosin (H&E). Images were taken with an Axio Imager.A2 upright microscope coupled to an AxioCam HR camera.

### Apoptosis assay

Terminal deoxynucleotidyl transferase-mediated dUTP nick end labelling (TUNEL) assays were performed on EB cryosections with an *In Situ* Cell Death Detection Kit, TMR Red (Roche), according to the manufacturer's protocol.

## Results

### Generation of ZO-1 and ZO-2 gene knockouts in mouse embryonic stem cells

**Figure 1:**
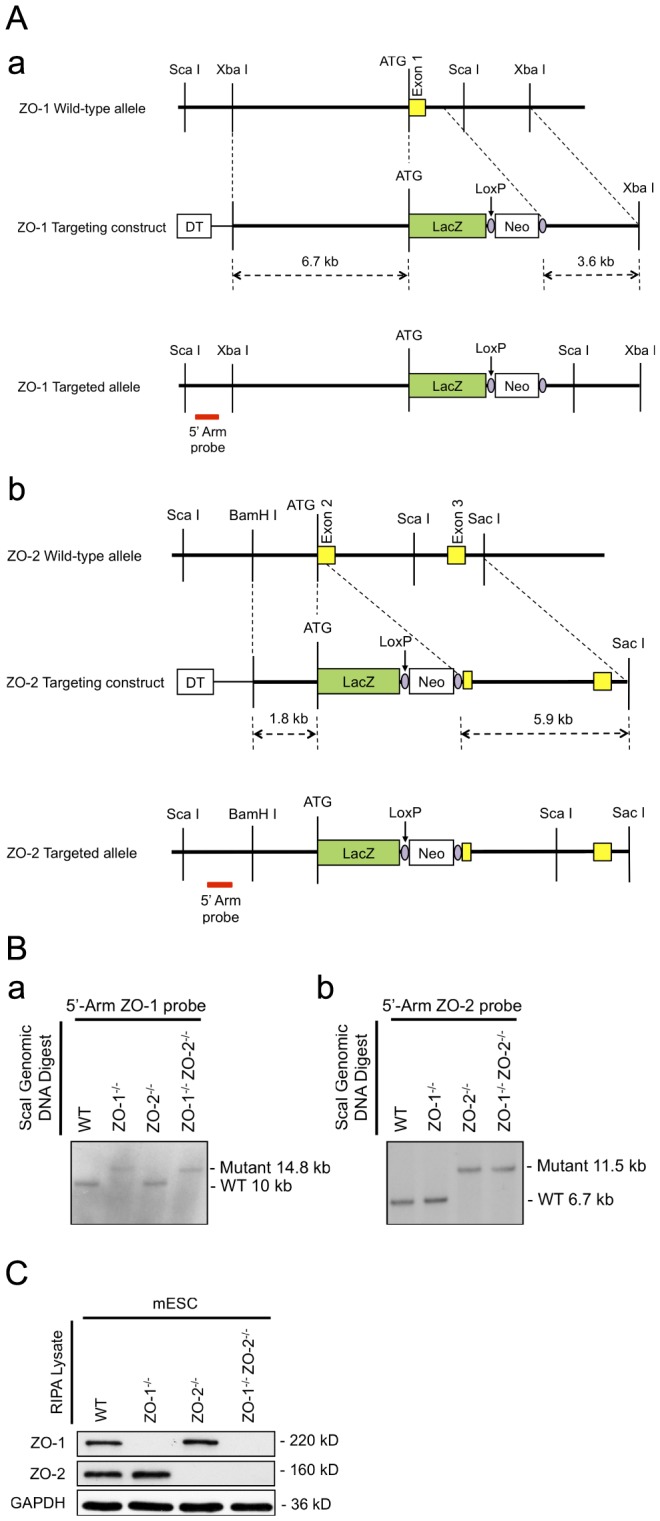
Targeting of the ZO-1 and ZO-2 locus and genotyping. (**A**) Targeting strategy. Schematic representation of the genomic loci restriction maps of ZO-1 showing exon 1 (panel a) and ZO-2 showing exons 2–3 (panel b) in yellow boxes with the initiation ATG. Through the in-frame insertion of a LacZ gene (green box) and a loxP-flanked (purple circle) Neo cassette (white box) immediately downstream of the ATG codon, the ZO-1 and ZO-2 allele-targeting constructs were designed to delete the entire ZO-1 exon 1 and part of the downstream intron; and part of ZO-2 exon 2 respectively. The red bar indicates the position of probe hybridization for Southern blot analysis. (**B**) Genotypic analysis by Southern blotting. ScaI-digested genomic DNA of selected mESC clones were hybridized with a DIG-labelled 5′ genomic DNA probe for the identification of homologous recombinants. 10 kb and 14.8 kb probe-hybridized fragments correspond to WT and targeted allele of ZO-1 locus respectively (panel a), whereas WT and targeted allele of ZO-2 locus are respectively represented by 6.7 kb and 11.5 kb fragments (panel b). (**C**) Protein expression analysis. mESC lysates were subjected to immunoblotting with anti-ZO-1 and anti-ZO-2 antibodies. The presence of ZO-1 and ZO-2 protein is indicated by 220 kD and 160 kD bands respectively. GAPDH served as a control for equal lysate input.

The ZO-1 (Fig. 1A, panel a) or ZO-2 (Fig. 1A, panel b) gene locus was targeted in W4 mESCs with a β-galactosidase gene (LacZ) knock-in targeting vector using the strategy as previously described [19] [26] and illustrated in Fig. 1A. Homozygous ZO-1 and ZO-2 double gene-deleted mESCs (ZO-1^-/-^ ZO-2^-/-^) were generated from the targeting of the ZO-2 locus of two independent ZO-1^-/-^ mESC clones. Targeted mESCs were selected in G418 and screened for homologous recombination at the ZO-1 and/or ZO-2 gene locus by Southern blot hybridization of ScaI-digested genomic DNA with gene-specific 5′ Arm probes (Fig. 1B, panels a and b). The 5′-Arm ZO-1 probe detected a 10 kb and 14.8 kb band corresponding to the ZO-1 WT and mutant alleles respectively. Only the ZO-1^-/-^ and ZO-1^-/-^ ZO-2^-/-^ mESC ScaI-digests were positive for the ZO-1 mutant but not WT allele, whereas the reverse was seen with the WT and ZO-2^-/-^ mESCs (Fig. 1B, panel a). The ZO-2 WT and mutant alleles were detected as 6.7 kb and 11.5 kb bands by the 5′-Arm ZO-2 probe. ZO-2 mutant but not WT alleles were identified in ZO-2^-/-^ and ZO-1^-/-^ZO-2^-/-^ mESCs. Conversely, only ZO-2 WT alleles were observed in WT and ZO-1^-/-^ mESCs (Fig. 1B, panel b). Western blot analysis validated the genotype results, showing an absence of both ZO-1 and ZO-2 proteins in ZO-1^-/-^ ZO-2^-/-^ mESCs, but absence of only ZO-1 protein in ZO-1^-/-^ and ZO-2 protein in ZO-2^-/-^ mESCs (Fig. 1B, panel c), thus indicating the successful targeting of the ZO-1 and/or ZO-2 genes.

### ZO-1^-/-^ ZO-2^-/-^ embryoid bodies fail to assemble TJs at the extraembryonic endoderm

**Figure 2:**
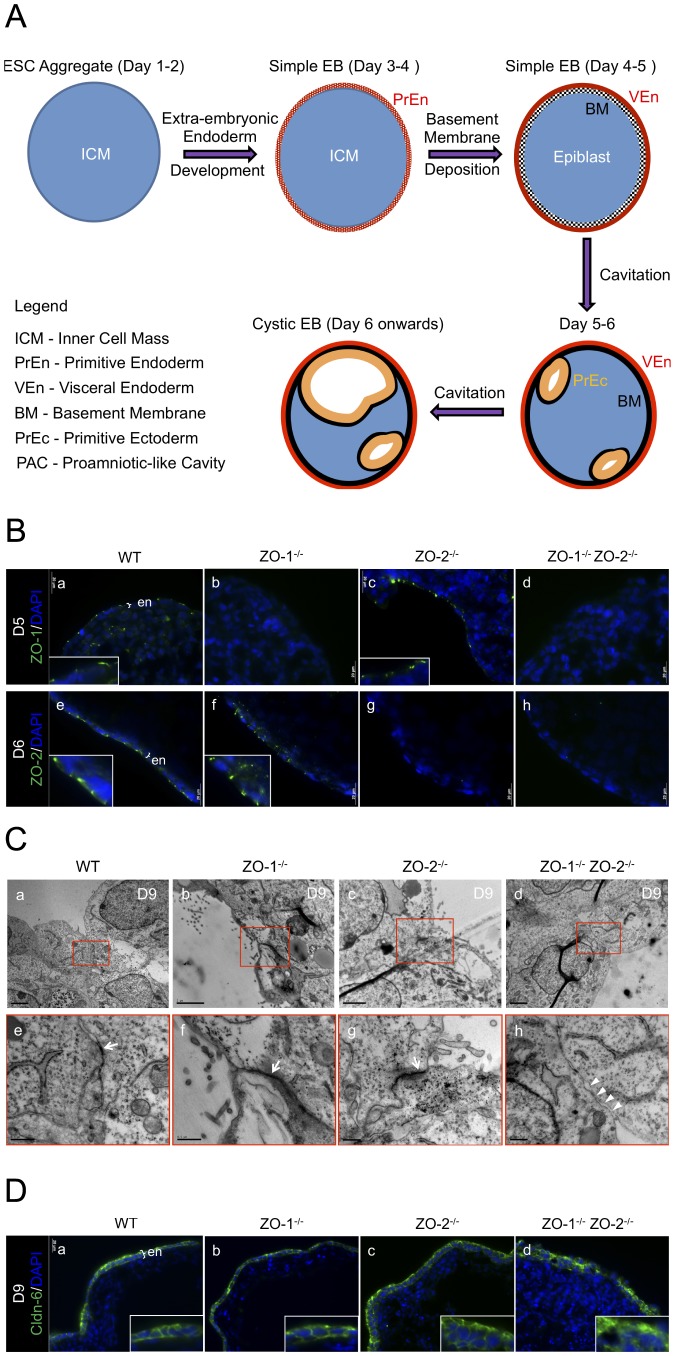
EB development and TJ formation. (**A**) Schematic diagram of EB development. The development of EBs starts with the aggregation of ESCs into suspended spheroids of inner cell mass (ICM) and parallels the development of the mouse blastocyst ICM. By day 3 of culture, the outer cells differentiate into a circumferential epithelium known as the primitive endoderm (PrEn). The apical and basal domains of the PrEn face the exterior environment and interior ICM core of the EB respectively. The EBs at this point are called simple EBs. Progressively from day 4 onwards, the PrEn basally secretes key ECM components Laminins and Collagen IV to form a basement membrane (BM). The PrEn also further differentiates in the visceral endoderm (VEn). In conjunction with this, the interior ICM differentiates into the epiblast. By day 5 or 6, the epiblast in contact with the BM in turn differentiates into the primitive ectoderm (PrEc) or epiblast epithelium. The rest of the epiblast not in contact with the BM will undergo apoptosis. The apoptotic bodies are removed by autophagy-initiated phagocytosis, leaving a progressively enlarging lumen or Proamniotic-like cavity (PAC) surrounded by the apical domain of the PrEc and the basal BM. EBs at this stage are called cystic EBs and equivalent to the egg cylinder stage of the mouse peri-implantation embryo just before gastrulation. (**B**) Immunofluorescence staining of ZO-1 and ZO-2 in Day-5 or -6 EB cryosections. WT (panels a and e), ZO-1^-/-^ (panels b and f), ZO-2^-/-^ (panels c and g) and ZO-1^-/-^ ZO-2^-/-^ (panels d and h) EBs were stained with antibodies to ZO-1 (panels a-d, green color) or ZO-2 (panels e-h, green color). Nuclei are labeled with DAPI (blue color). Magnification of image in insets. ExEn is indicated here as ‘en’. (**C**) Transmission electron micrographs. TJ complex at the apico-lateral membrane of Day-9 EB ExEn was visualized by TEM as electron-dense material (indicated by arrows in magnification of inset) in WT (panels a and e), ZO-1^-/-^ (panels b and f) and ZO-2^-/-^ (panels c and g) EBs. This was absent in ZO-1^-/-^ ZO-2^-/-^ EBs (panels d and h, arrowheads in magnification of inset). (**D**) Immunofluorescence staining of Cldn-6 in Day-9 EB cryosections. WT, ZO-1^-/-^, ZO-2^-/-^ and ZO-1^-/-^ ZO-2^-/-^ EBs were stained with antibodies to Cldn-6 (panels a–d, green color). Nuclei are labeled with DAPI (blue color). Magnification of image in insets. ExEn is indicated here as ‘en’.

To investigate the functional roles of ZO-1 and ZO-2 in epithelial morphogenesis and *de novo* TJ biogenesis, mESCs were cultured into EBs. The development of EBs is illustrated and described in Fig. 2A.

ZO protein staining at the differentiated outer layer extraembryonic endoderm (ExEn, denoted as ‘en’ in image), i.e. primitive endoderm (PrEn) and visceral endoderm (VEn), was investigated (Fig. 2B). Immunofluorescence staining of ZO-1 and ZO-2 proteins displayed apico-lateral localization on the ExEn of WT EBs (Fig. 2B, panels 〈 and e). This was not observed in ZO-1^-/-^ ZO-2^-/-^ EBs (Fig. 2B, panels d and h). As expected, ZO-1 localization was absent in ZO-1^-/-^ (Fig. 2B, panel b) but present in ZO-2^-/-^ EBs (Fig. 2B, panel c). Conversely, ZO-2 staining was not detected in ZO-2^-/-^ (Fig. 2B, panel g) but found in ZO-1^-/-^ EBs (Fig. 2B, panel f). These results indicate that the apico-lateral localization of ZO-1 and ZO-2 proteins in the epithelium is independent of each other.

We next used transmission electron microscopy (TEM) to examine ExEn TJ assembly in EBs cultured for 9 days (Fig. 2C, panels a–h). Electron-dense material, consistent with TJ plaques, was present at the most apico-lateral region between adjacent ExEn cells of WT, ZO-1^-/-^ and ZO-2^-/-^ EBs (Fig. 2C, panels a-c, red inset, and panels e-g, arrows). The intercellular space at this area appears obliterated, typical for TJ ultrastructure. In contrast, ZO-1^-/-^ ZO-2^-/-^ EBs exhibited no electron-dense material, suggesting a deficiency in the assembly of the TJ plaque. Furthermore, this area appears to have an expanded intercellular space (Fig. 2C, panel d, red inset, and panel h, arrowheads).

Since Claudins (Cldn) are the key constituent of TJ strands, their localization was studied to complement the TEM observations. Cldn-6 was chosen as a representative of the Cldn family since it is an EB early epithelialization marker [28]. In agreement with the TEM data, Cldn-6 distinctly immunostained the lateral membrane of the ExEn epithelium of WT, ZO-1^-/-^ and ZO-2^-/-^ EBs (Fig. 2D, panels a–c). However, this staining was diffused at the membrane periphery in ZO-1^-/-^ ZO-2^-/-^ EBs (Fig. 2D, panel d), inferring a lack of junctional localization.

Collectively, these data reveal that the absence ZO-1 and ZO-2 results in the inability of the EB ExEn epithelium to assemble TJ, whereas the sole deletion of either gene does not alter TJ formation.

### The extraembryonic endoderm is disorganized and abnormally permeable in ZO-1^-/-^ ZO-2^-/-^ embryoid bodies

**Figure 3:**
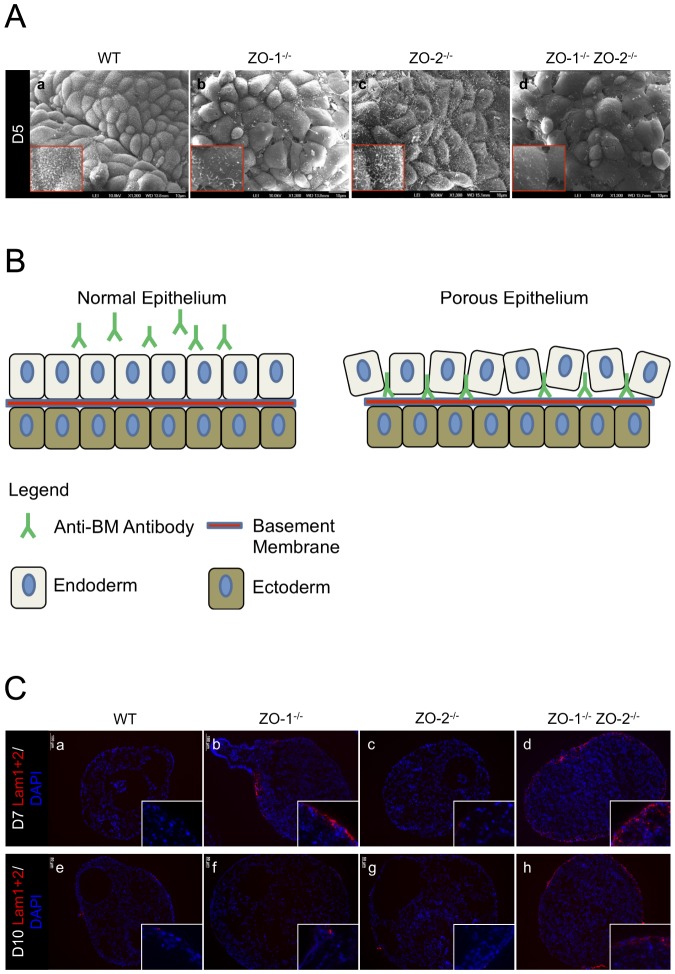
ExEn organization and permeability is abnormal in EBs lacking ZO-1 and ZO-2. (**A**) Scanning electron micrographs. ExEn apical surface morphology of WT control and ZO gene-deleted EBs was analyzed by SEM. The apical surface of compacted ExEn cells can be seen covered with dense hair-like microvilli in WT (panel a) and ZO-2^-/-^ (panel c) EBs. ZO-1^-/-^ (panel b) and ZO-1^-/-^ ZO-2^-/-^ (panel d) EB ExEn cells are more loosely packed and bear sparse microvilli formation. Magnification of image in insets. (**B**) ExEn permeability assay illustration. Intact ExEn of live EBs pre-incubated with anti-BM IgG will not allow penetration of the antibodies to the underlying BM. In a permeability-compromised ExEn, the anti-BM IgG can move across the ExEn paracellularly and bind to the underlying BM. (**C**) Permeability assay using anti-Lam1+2 pre-incubate. Live WT, ZO-1^-/-^, ZO-2^-/-^ and ZO-1^-/-^ ZO-2^-/-^ EBs at Day-7 (panels a-d) and Day-10 (panels e–h) of culture were pre-incubated for 2 hrs in medium spiked with IgG recognizing the BM component Laminin1+2. The EBs were subsequently washed and fixed, and the embedded sections were then permeabilized and treated with fluorophore-tagged secondary antibodies to visualize the anti-Lam1+2 IgG (red color). Nuclei are labeled with DAPI (blue color). Magnification of image in insets.

Through the observations of TEM and IF vertical EB sections, we noticed an irregular morphology of the ExEn layer in ZO-1^-/-^ ZO-2^-/-^ EB sections compared to WT control. To explore in detail if the ZO gene-deletions have an effect on ExEn morphology, we visualized the ExEn apical surface using scanning electron microscopy (SEM) at different time points of culture (Fig. 3A). At 5 days of EB culture, WT and ZO-2^-/-^ ExEn cells were arranged in a compact epithelial cobblestone organization and carpeted densely with apical surface microvilli protrusions (Fig. 3A, panels a and c). In contrast, the entire surface of the ExEn layer of ZO-1^-/-^ ZO-2^-/-^ EBs was disorganized and discontiguous, with cell-cell arrangement lacking compactness and boundaries showing obvious gaps. Furthermore, the apical cell surface looked distended and microvilli were sparsely present on this surface (Fig. 3A, panel d). This phenotype was also observed in ZO-1^-/-^ EBs, albeit to a lesser extent, with patches of disorganized cells amidst regularly ordered ones (Fig. 3A, panel b). Interestingly, the irregular surface of ZO-1^-/-^ EB recovered progressively to normalcy by Day-10 of culture, displaying the usual contiguous cobblestone cell arrangement. However, ZO-1^-/-^ ZO-2^-/-^ EBs still exhibited extensive ExEn disorganization throughout the culture period (Fig. S1A and B).

Since the ExEn epithelial cell layer of ZO-1^-/-^ ZO-2^-/-^ and, to an extent, ZO-1^-/-^ EBs are loosely packed and have obvious cell-cell boundary gaps, we next tested if the ExEn permeability barrier is compromised. To study this, we devised a simple assay in which live EBs where pre-incubated with anti-basement membrane (BM) antibodies added to the culture medium for 2hrs. Since these antibodies detect basement membrane components, a normal functioning permeability barrier would not be expected to allow IgG to penetrate the ExEn paracellularly, especially since IgG has a large molecular weight of 150 kD [29]. However, if as suspected from the SEM analysis the barrier function is compromised, a large solute like IgG could possibly move paracellularly through the ExEn, reach the underlying BM and, in the case of a specific anti-BM IgG, bind to it. Here, after washing off unbound anti-BM IgG, followed by fixation and permeabilization of the EBs, the anti-BM can then be visualized with fluorophore-tagged secondary antibodies (Fig. 3B).

This permeability assay was carried out by pre-incubating EBs with antibodies against BM components Laminin (Lam1+2) (Fig. 3C) or Perlecan (PLC) (Fig. S3). In Day-7 (Fig. 3C, panels a-d) and Day-10 EB cultures (Fig. 3C, panels e-h), except for a few non-specific residual spots of stain fixed on the ExEn surface, no anti-Lam1+2 IgG penetration was detected at the BM underlying the ExEn of WT (Fig. 3C, panels a and e) and ZO-2^-/-^ EBs (Fig. 3C, panels c and g). However, at Day-7, some regions at the ZO-1^-/-^ EB periphery were stained with anti-Lam1+2 IgG (Fig. 3C, panel b). This staining was unlike the residual spots seen in WT and ZO-2^-/-^, but penetrated a few cells deeper into the periphery and stained only the extracellular region where the BM would be secreted, thus signifying a breach in ExEn paracellular permeability barrier. This staining was even more pronounced and extensive in ZO-1^-/-^ ZO-2^-/-^ EBs, with anti-Lam1+2 IgG infiltrating pass the ExEn throughout the entire circumference of the EB (Fig. 3C, panel d). This indicated a severely compromised permeability barrier in ZO-1^-/-^ ZO-2^-/-^ EBs, which was still observed in Day-10 cultures (Fig. 3C, panel h). However, by this time, the anti-Lam1+2 IgG was no longer able to stain the BM in ZO-1^-/-^ EBs, suggesting the restoration of a normal ExEn permeability barrier by Day-10 (Fig. 3C, panel f). Similar results were obtained with EB pre-incubation in anti-PLC IgG (Fig. S3), and are in line with the ExEn surface organization SEM data.

In conclusion, SEM and permeability assay studies on EB ExEn organization and integrity show that the absence of both ZO-1 and ZO-2 drastically compromise the paracellular permeability barrier of the ExEn layer, concurrent with less compact organization of ExEn cells. These phenotypes were intermediate in EBs lacking only ZO-1, especially at early stages (Day-5 to -7) of EB culture, but normalcy was restored at later stages (Day-10).

### Extraembryonic endoderm differentiation is normal in embryoid bodies lacking ZO-1 and/or ZO-2

Although the mutant phenotypes observed in ZO-1^-/-^ ZO-2^-/-^ EB ExEn can be corroborated by similar studies done in immortalized epithelial cell-lines [16] [21] [17], the EB and immortalized epithelial cell-line models are fundamentally different. Epithelial cell-lines are already differentiated when experimentally manipulated, whereas the ExEn of EBs differentiate into epithelia from non-polarized pluripotent inner cell mass (ICM) cells through an inbuilt developmental mechanism. Since we previously reported that ZO-1 affects self-renewal and differentiation of mESCs [26], it was important to determine if the absence of ZO-1 in mESCs could impede the differentiation of EB ICM cells into the ExEn epithelium and thus mistakenly lead to the interpretation of supposed ExEn abnormality. We therefore next examined the differentiation of EBs into outer layer primitive and visceral endoderm using molecular markers characteristically expressed at different stages of ExEn differentiation. GATA-4 and -6, Dab2 and Troma-1 were used as ExEn markers. GATA-4 and -6 are transcription factors that are expressed in the PrEn, early in its differentiation from the ICM. Dab2 is a downsteam target of GATA-6 and is expressed in the PrEn and subsequent VEn. These three proteins are essential for the formation of the PrEn on the outer EB layer [30]. The intermediate filament Keratin-8, recognized by the antibody Troma-1, is an epithelial cell marker and is present in early PrEn differentiation [31].

**Figure 4:**
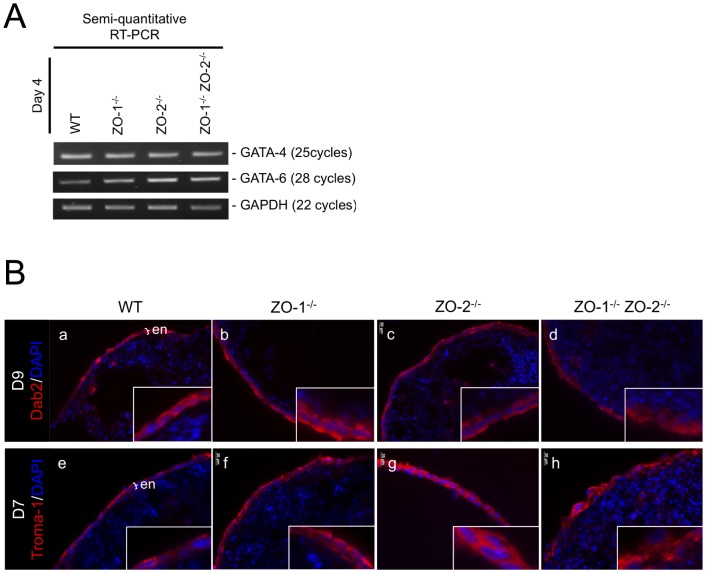
EB ExEn differentiation is not affected by ZO-1 and/or ZO-2 deletion. (**A**) Semi-quantitative RT-PCR of Day-4 EB. Reverse transcribed cDNA was amplified with specific GATA-4 and -6 primer sets at optimized cycle numbers (indicated on right side of panels). GAPDH amplification served as a control for equal RNA input. (**B**) Immunofluorescence staining of EB ExEn. Fixed and permeabilized EB cryosections were treated with antibodies immunoreactive to Dab2 (panels a-d) and Keratin-8 (Troma-1) (panels e-h) and visualized (red color). Nuclei are labeled with DAPI (blue color). Magnification of image in insets. ExEn is indicated here as ‘en’.

The presence of GATA-4 and -6 mRNA transcripts in EBs of Day-4 culture were investigated by RT-PCR (Fig. 4A). Day 4 EBs were chosen for this semi-quantitative study as the PrEn would have differentiated by this time. After optimizing the PCR cycle number to coincide with the exponential phase of amplification, the amplicon intensity between WT and the three null mutants for both GATA-4 and -6 amplifications were indistinguishable. The GATA-6 downstream target gene, Dab2 was next tested for expression at the protein level in the ExEn by immunostaining (Fig. 4B, panels a–d). Dab2 was detected in the cytoplasm of the ExEn of all three ZO null mutants and the WT control EBs. Similarly, Keratin-8 (Troma-1) was present in all EB types (Fig. 4B panels e–h), thus indicating that the ExEn layer is indeed differentiated and is of epithelial cell-type in the EBs lacking ZO proteins.

Taken together, the data shown here indicate that the absence of ZO-1 and/or ZO-2 do not affect the differentiation of the ExEn from the EB ICM cells and that the ExEn is an epithelium. Thus, the above described effects on epithelial properties in the ExEn of ZO-1^-/-^ and ZO-1^-/-^ ZO-2^-/-^ EBs are not due to a failure of epithelial differentiation.

### Deletion of ZO-1 and ZO-2 represses the expression of Podocalyxin and impedes the apical localization of Ezrin in the extraembryonic endoderm

**Figure 5:**
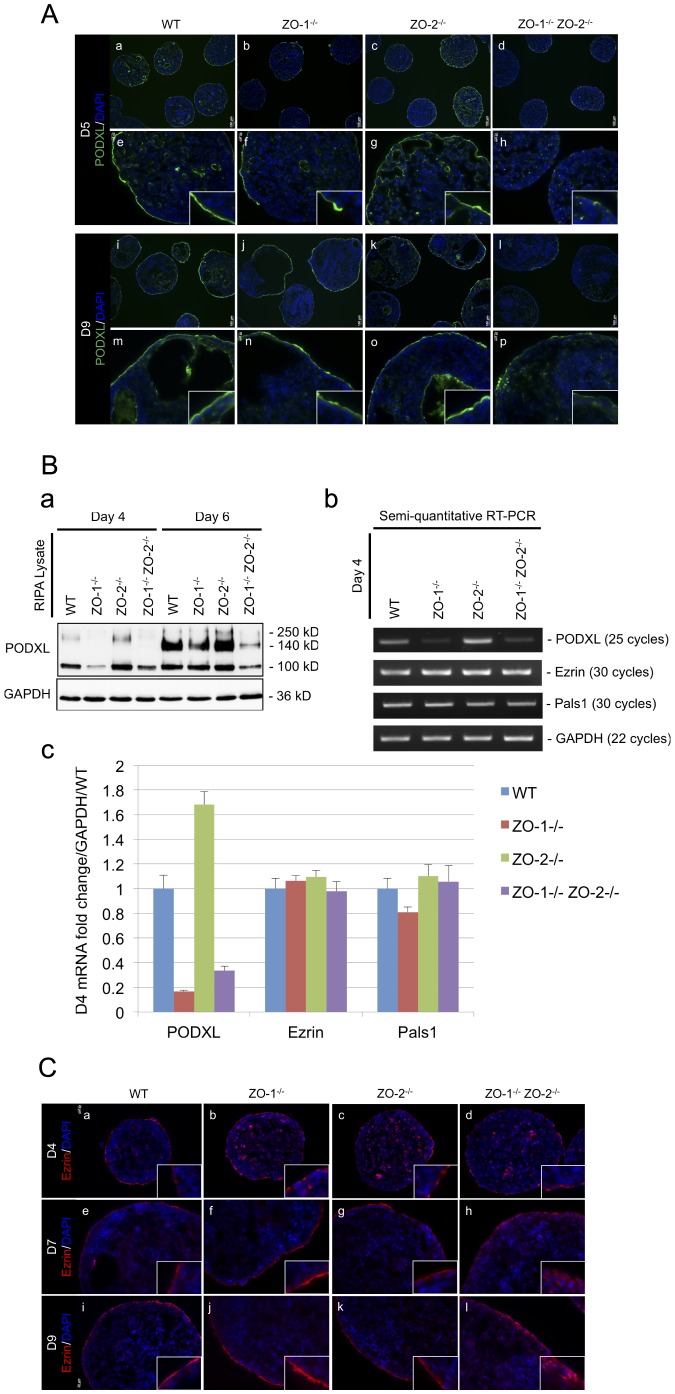
PODXL expression and Ezrin localization at the ExEn is aberrant when ZO-1 and ZO-2 is deleted. (**A**) Immunofluorescence staining of PODXL. EB cryosections from Day-5 (panels a–h) and -9 (panels i–p) cultures were immunostained for PODXL (green color). Nuclei are labeled with DAPI (blue color). Magnification of image in insets. (**B**) PODXL expression. Protein expression of PODXL at Day-4 and -6 was determined by immunoblot. 100 (immature glycosylated), 140 (mature glycosylated) and 250 (dimer) kD bands represent the various post-translationally modified forms of PODXL. GAPDH was used as a lysate loading control (panel a). PODXL transcript level was analyzed by semi-quantitative RT-PCR. Reverse transcribed cDNA was amplified with specific primer sets at optimized cycle numbers (indicated on right side of panels). Ezrin and Pals1 were selected as epithelia polarized controls. GAPDH amplification served as a control for equal RNA input (panel b). Quantitative real-time PCR was also employed to validate PODXL transcript expression. Expression levels are presented as average fold-change of three separate experiments normalized to GAPDH and relative to WT control (panel c). (**C**) Immunofluorescence staining of Ezrin. Cryosections of EBs harvested at Day-4 (panels a–d), Day-7 (panels e–h) and Day-9 (panels i–l) of culture were immunostained with antibodies against Ezrin (red color). Nuclei are labeled with DAPI (blue color). Magnification of image in insets.

Podocalyxin (PODXL) is a single-pass transmembrane sialoglycoprotein that was first discovered in specialized epithelial cells of the renal glomerulus (podocytes) [32]. It is localized at the apical membrane of polarized epithelial cells and has a heavily sialylated ectodomain and a cytosolic tail that associates indirectly with actin filaments [33]. Since PODXL positively regulates the formation of microvilli [34] and the apical domain [35] in epithelial cells, we investigated if ZO deficiency had an effect on this protein. EBs were monitored for PODXL expression and localization continually over a 9 day period of culture by immunofluorescence microscopy. At Day-5, WT and ZO-2^-/-^ EBs (Fig. 5A, panels a and e; panels c and g, respectively) exhibited an accumulation of PODXL at the apical membrane of the entire ExEn layer. This staining could also be seen on the apical membrane of the primitive ectoderm (PrEc) surrounding nascent cavities at the EB interior. However, in ZO-1^-/-^ EBs (Fig. 5A, panels b and f), apical membrane PODXL staining was not continuous along the entire ExEn layer, with some areas of the ExEn lacking PODXL. This anomaly was even more pronounced with ZO-1^-/-^ ZO-2^-/-^ EBs (Fig. 5A, panels d and h), where apical membrane PODXL concentration was fragmented and faint throughout the ExEn layer. As with the restoration to normalcy of the ExEn cell organization and permeability function reported earlier, the ZO-1^-/-^ ExEn layer displayed a continuous apical membrane staining at later time points of culture, as depicted at Day-9 (Fig. 5A, panels j and h). This staining was indistinguishable from the normal PODXL apical membrane enrichment in WT and ZO-2^-/-^ ExEn at Day-9 (Fig. 5A, panels i and m; panels k and o, respectively). As expected, apical membrane PODXL staining of ZO-1^-/-^ ZO-2^-/-^ ExEn remained faint and discontinuous even at Day-9 (Fig. 5A, panels l and p), indicating an inability for recovery to normalcy.

Since the abnormal PODXL ExEn was manifested as faint and discontinuous apical membrane staining without any observable mislocalization, we suspected that this might be due to a compromised PODXL expression. Therefore, we next examined both protein and mRNA transcript expression of PODXL (Fig. 5B). Day-4 and Day-6 EB lysates were analyzed for PODXL protein expression (Fig. 5B, panel a). PODXL expression can be detected on immunoblots as post-translationally modified forms of 100 (immature glycosylated), 140 (mature glycosylated) and 250 (dimer) kD bands [36]. The immature glycosylated PODXL (100 kD) was the predominant form expressed at Day-4 in WT and all ZO null mutants. This expression was considerably reduced in ZO-1^-/-^ and ZO-1^-/-^ ZO-2^-/-^ EB lysates at Day-4 compared with WT and ZO-2^-/-^ EB levels. By Day-6, the expression of mature glycosylated PODXL (140 kD) starts to appear and predominate. At this time, ZO-1^-/-^ EB PODXL protein levels start to increase and match WT and ZO-2^-/-^ EB levels, but ZO-1^-/-^ ZO-2^-/-^ levels remained significantly lower, consistent with the immunostaining observations.

As repressed proteins levels could be a result of transcriptional regulation, we looked at the expression of PODXL mRNA transcript. Since we were also interested to evaluate if the changes in PODXL expression were specific for PODXL or a general phenomenon affecting other membrane localized proteins, we looked at two additional membrane polarized epithelial proteins, Ezrin [37] and Pals1 [38], as indicators. Semi-quantitative RT-PCR was carried out on Day-4 EBs at optimized cycle numbers (Fig. 5B, panel b). Intriguingly, PODXL transcript levels were repressed in ZO-1^-/-^ and ZO-1^-/-^ ZO-2^-/-^ EBs compared with WT EBs, whereas, as expected, ZO-2^-/-^ EB levels were similar to WT EBs. Notably, Ezrin and Pals1 transcript levels were similar among the WT and all three ZO null mutant EBs. This repression of PODXL transcript expression was further validated using quantitative real-time PCR (Fig. 5B, panel c). As anticipated, the qPCR data matched that of RT-PCR, showing at least a four-fold decrease in ZO-1^-/-^ and ZO-1^-/-^ZO-2^-/-^ EB PODXL transcript levels compared with WT EBs. Interestingly, at both protein (Fig. 5B, panel a) and transcript levels (Fig. 5B, panels b and c), ZO-2^-/-^ EBs appeared to express slightly more PODXL compared with WT EBs, especially at the earlier Day-4 time point. We suspect this is due to the marginally earlier formation of cavity-lined PrEc in ZO-2^-/-^ EBs (Fig. 5A, panel g), which, together with the ExEn, will contribute to the total PODXL expression level, thus elevating it above the later-cavitating WT EBs (Fig. 5A, panel e).

Ezrin is a member of the Ezrin-Radixin-Moesin (ERM) family of submembrane localized proteins, which function in the organization of specialized membrane domains [39]. In polarized epithelial cells, Ezrin localizes at the sub-apical membrane where it interacts directly or indirectly with integral membrane proteins, cross-linking them with the underlying cortical actin network. Since Ezrin can regulate microvilli formation, like PODXL [40], we investigated its localization in the EB ExEn. Although there was no change in Ezrin expression in ZO-1^-/-^ ZO-2^-/-^ EBs (Fig. 5B, panels b and c), we suspected that Ezrin membrane localization might be affect by the downregulation of PODXL levels, since Ezrin can interact with -and be membrane-recruited by- PODXL [41] [42]. Ezrin localization was visualized in EBs by immunostaining at Day-4, -7 and -9 of culture (Fig. 5C). At Day-4 (Fig. 5C, panels a-d), Ezrin started to accumulate at the apical membrane of the ExEn layer, appearing as fragmented apical staining. By Day-7 and -9, this staining was continuous along the entire apical surface of the ExEn in WT, ZO-1^-/-^ and ZO-2^-/-^ EBs (Fig. 5C, panels e–g and i–k, respectively). Strikingly, in ZO-1^-/-^ ZO-2^-/-^ EBs, Ezrin failed to localize to the ExEn apical membrane at both Day-7 and -9 (Fig. 5C, panels h and l, respectively), and displayed a diffused cytosolic localization. This was coincident with the downregulated PODXL expression (Fig. 5A, panels l and p) and diffused cytosolic Cldn-6 localization (Fig. 2D, panel d) at the ExEn of Day-9 ZO-1^-/-^ ZO-2^-/-^ EBs.

Collectively, we have revealed that the absence of ZO-1 and ZO-2 is associated with a downregulation of PODXL expression at both the protein and transcript level. This depletion of PODXL expression likely leads to the mislocalization of the sub-apical membrane protein Ezrin, possibly due to lack of membrane recruitment by PODXL, and the disorganization of the ExEn layer.

### ZO-1 and ZO-2 deletion leads to the aberrant deposition of basement membrane and cavitation defects

**Figure 6:**
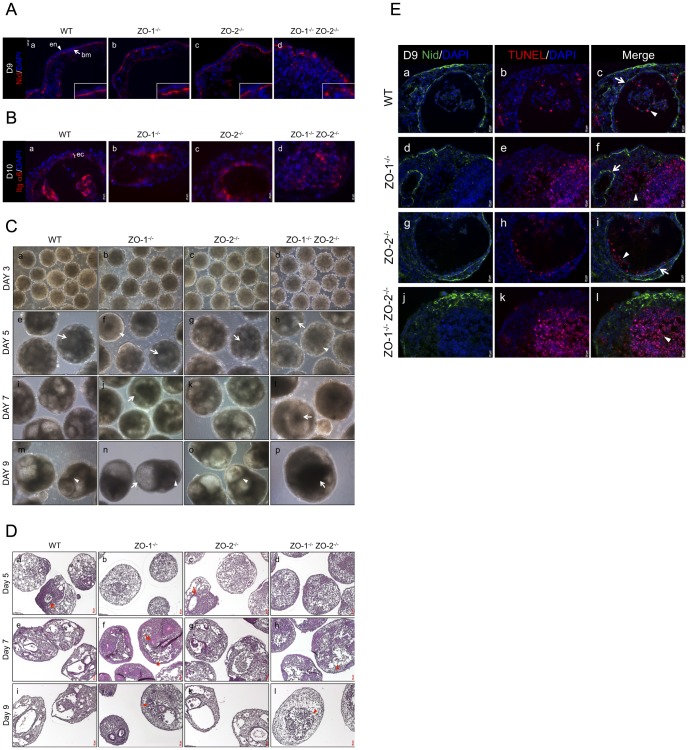
Depletion of ZO-1 and ZO-2 is associated with aberrant basement membrane deposition and irregular cavitation. (**A**) Immunofluorescence staining of Nidogen. EB cryosections from Day-9 cultures (panels a–d) were immunostained for the BM component Nidogen (red color, arrow). ExEn and BM are indicated by arrowhead and arrow respectively. Nuclei are labeled with DAPI (blue color). Magnification of image in insets. (**B**) Immunofluorescence staining of PrEc. The immunodetection of Integrin α6 was used as a specific marker for PrEc visualization in Day-10 EB cultures (panels a–d, red color). Nuclei are labeled with DAPI (blue color). PrEc is indicated here as ‘ec’. (**C**) Phase-contrast microscopy. Cavity development in live EBs was tracked by phase-contrast imaging on Day-3 (panels a–d), Day-5 (panels e–h), Day-7 (panels i–l) and Day-9 (panels m–p) of culture. (**D**) H&E histological staining. PFA-fixed and paraffin-embedded EB sections were treated with H&E stains to visualize the gross structure of the EBs. EB histology was analyzed at Day-5 (panels a–d), Day-7 (panels e–h) and Day-9 (panels i–l). (**E**) TUNEL staining of apoptotic cells. Cryosections of EBs at Day-9 culture were immunostained with Nidogen (Nid) to demarcate the BM boundary of cavities (panels a,d,g,j, green color). TUNEL-positive apoptotic cells were visualized in red color (panels b,e,h,k). Merged images (panels c,f,i,l). Nuclei are labeled with DAPI (blue color).

A hallmark of epithelial morphogenesis is the deposition of a basal basement membrane (BM). In EBs, key BM components like Laminins and Collagen IV are secreted by the ExEn and deposited as a basal underlying band [43]. As the ExEn layer is disorganized in the ZO-1^-/-^ ZO-2^-/-^ EBs, we investigated if this had an effect on the deposition of the BM. The BM consists of various components, including Laminins, Collagen, Perlecan and Nidogen [44], with Laminins playing a key role in assembly of the BM [45]. Immunofluorescence staining of Nidogen (Nid) (Fig. 6A), and Perlecan, Collagen IV and Laminin 1+2 (Fig. S4), was done on Day-9 and Day-12 cultures, respectively, when BM deposition in WT control EBs was complete. At Day-9, Nidogen (denoted as ‘bm’ in image) is visible as a continuous band underlying the ExEn in WT, ZO-1^-/-^ and ZO-2^-/-^ EBs (Fig. 6A, panels a–c). In contrast, in ZO-1^-/-^ ZO-2^-/-^ EBs, the BM was instead irregularly deposited and looked discontinuous and fragmented (Fig. 6A, panel d), probably due to the disorganization of the ExEn. This same pattern of BM deposition was also observed in Day-12 EB cultures stained for the other BM components (Fig. S4).

The BM is not merely a foundational support that underpins the epithelium but also regulates diverse biological processes like differentiation and survival via cell-surface receptors such as integrins [46]. During EB development, the BM regulates the formation, polarization and survival of the primitive ectoderm (PrEc) layer [47] [48]. We therefore next determined if the formation of PrEc was influenced by the abnormal BM deposition in ZO-1^-/-^ ZO-2^-/-^ EBs. At Day-10 culture, Integrin α6 (Itg α6), a specific marker for the PrEc [49], can be seen as a contiguously arranged ring of organized epithelium (denoted as ‘ec’ in image) surrounding a cavity in WT, ZO-1^-/-^ and ZO-2^-/-^ EBs (Fig. 6B, panels a–c). Intriguingly, the PrEc was unsuccessfully formed in ZO-1^-/-^ ZO-2^-/-^ EBs (Fig. 6B, panel d), displaying an amorphous mass of small Itg α6-positive cells devoid of epithelial morphology, often containing condensed and fragmented nuclei, characteristic of apoptosis [50]. These manifestations imply that the fragmented deposition of BM in ZO-1^-/-^ ZO-2^-/-^ EBs results in failure of PrEc cells to arrange into an epithelium and instead these cells undergo apoptosis.

In our null mutants, the cavities in ZO-1^-/-^ ZO-2^-/-^ EBs were often enlarged and had dense material within the lumen, which gradually accumulated to fill up the cavities. To study this phenomenon in greater detail, phase-contrast imaging (Fig. 6C) and Hematoxylin and Eosin (H&E) histological staining (Fig. 6D) were employed to track EB development. Phase-contrast microscopy of live EBs at various time points indicated no discernible morphological difference between the WT control and null mutants at Day-3 (Fig. 6C, panels a–d) of EB culture. Small nascent cavities (arrows) began to form at Day-5 (Fig. 6C panels e–h) for all the EBs. However, cavitation was delayed in some EBs of the ZO-1^-/-^ and ZO-1^-/-^ ZO-2^-/-^ null mutants (arrowheads). Cavities progressively enlarged into a round lumen in WT and ZO-2^-/-^ EBs and were surrounded by an obvious PrEc layer (arrowhead), as depicted at Day-7 and -9 (Fig. 6C, panels i,k and m,o, respectively). In contrast, at Day-7, the cavities of ZO-1^-/-^ and ZO-1^-/-^ ZO-2^-/-^ EBs were irregularly shaped. ZO-1^-/-^ EBs appeared to cavitate along the EB periphery without an obvious surrounding PrEc (Fig. 6C, panel j, arrow). By Day-9 (Fig. 6C, panel n), a bubble-like cavity (arrow) was seen at the side of these EBs, while smaller cavities, often containing dense material (arrowhead), formed at the EB interior. The ZO-1^-/-^ ZO-2^-/-^ EBs usually had one large central cavity at Day-7, extending to the EB periphery. They often contained dense material (Fig. 6C, panel l, arrow) and had no obvious encircling PrEc. Surprisingly, by Day-9, this large cavity diminished in size due to the accumulation of dense material (Fig. 6C, panel p, arrow).

These phase-contrast observations were validated by H&E staining of PFA-fixed and paraffin-embedded sections. Similar to the phase-contrast observations, small nascent cavities formed at Day-5 WT and ZO-2^-/-^ EBs (Fig. 6D, panels a and c, arrows), but cavitation was delayed in ZO-1^-/-^ and ZO-1^-/-^ ZO-2^-/-^ null mutants. At Day-7, a few small PrEc-lined cavities were present in ZO-1^-/-^ EBs (Fig. 6D, panel f, arrow). In addition, extensive amorphously-shaped cavities without obvious PrEc were present, and this was also observed in ZO-1^-/-^ ZO-2^-/-^ EBs. These cavities appeared to contain an accumulation of cells with dense Hematoxylin-stained nuclei (dark blue) (Fig. 6D, panels f and h, arrowheads). By Day-9, the cavities of ZO-1^-/-^ ZO-2^-/-^ EBs appeared to be filled with these dense-nucleated cells and no clear lumen was noticeable, unlike ZO-1^-/-^ EBs, in which this accumulation was mitigated (Fig. 6D, panels j and l, arrowheads).

As we suspected that the accumulated dense-nucleated cells were apoptotic bodies, we fluorescent stained the EBs using the terminal deoxynucleotidyl transferase-mediated dUTP nick end labelling (TUNEL) assay to detect DNA fragmentation as an indicator of apoptosis. The BM component Nidogen (Nid) was used as a co-stain to demarcate the cavity boundary. Day-9 WT and ZO-2^-/-^ EBs (Fig. 6E, panels a–c and g–i, respectively) displayed large cavities surrounded by a BM-attached PrEc layer (arrow). The lumen was largely cleared except for a few residual TUNEL-positive apoptotic bodies (arrowhead). Smaller, cleared, PrEc-bound cavities were visible in ZO-1^-/-^ EBs (Fig. 6E panels d–f, arrow), in addition to partially cleared PrEc-absent regions containing apoptotic bodies (arrowhead). No obvious cavity was observed in ZO-1^-/-^ ZO-2^-/-^ EBs as expected. Instead, massive accumulation of apoptotic bodies was seen at the EB core (Fig. 6E panels j–l, arrowhead).

In conclusion, we have shown that coincident with the disorganization of the ExEn layer, BM deposition is abnormally fragmented and discontinuous in ZO-1^-/-^ ZO-2^-/-^ EBs. This is associated with a failure to form a contiguous PrEc layer, leading to the apoptosis of PrEc/epiblast cells in the EB interior, probably due to a lack of survival signals from the irregular BM. This apoptosis initially lead to the formation of extensive cavities without a PrEc-lining. However, there was a progressive accumulation of apoptotic bodies which were not cleared and eventually filled up the lumen, thus diminishing the cavity. ZO-1^-/-^ EBs displayed phenotypes intermittent to ZO-1^-/-^ ZO-2^-/-^ EBs and presented both small PrEc-lined cavities and partially cleared apoptotic areas.

## Discussion

The roles of ZO proteins have been widely studied in the context of tight junction (TJ) function. Studies in immortalized mammalian epithelial cell-line models primarily focused on TJ assembly and its selective permeability barrier function. These investigations have implicated both, ZO-1 and ZO-2, as vital regulators of TJ strand formation and paracellular transepithelial transport [16] [17]. Such findings may explain the mechanism behind the developmental arrest of ZO-1 depleted mouse morula [51] or the delayed blastocyst morphogenesis of ZO-1 and/or ZO-2 depleted mouse embryos [52]. These *in vitro* studies on morula-blastocyst development, and the study on self-renewal and differentiation of ZO-1^-/-^ mouse embryonic stem cells [26], point to the significance of the ZO proteins in early mammalian embryogenesis. This importance is reflected in the early embryonic lethality of ZO-1 and ZO-2 null mutant mice [19] [18].

To provide a clearer understanding and dissect the roles that ZO-1 and ZO-2 collectively or individually play in early embryogenesis, we employed the *in vitro* embryoid body (EB) model. Aside from being a relevant embryogenesis model due to its recapitulation of mouse peri-implantation embryonic morphogenesis, this system offers a more natural approach compared with conventional immortalized epithelial cell-line models. Unlike mature epithelial cell-lines, the EB cells can spontaneously differentiate into epithelium that deposits a basement membrane (BM) which influences the creation of a cavity. This is therefore suited for the study of *de novo* TJ formation and epithelial morphogenesis.

We report here that upon targeted deletion of ZO-1 and ZO-2 gene expression (ZO-1^-/-^ ZO-2^-/-^), the EB outer layer extraembryonic endoderm (ExEn) was devoid of TJ formations, as determined by transmission electron microscopy and Claudin-6 staining of EB mid-plane sections. The individual deletion of ZO-1 or ZO-2 did not have an apparent effect on TJ assembly, implying redundancy in their ability to regulate TJ formation. These observations are in agreement with what has been reported in the ZO-1 knockout and/or ZO-2 knockdown EpH4 mouse mammary epithelial cell-lines [53] [16]. Interestingly, however, the lack of TJs in ZO-1^-/-^ ZO-2^-/-^ EB ExEn was associated with gross surface morphological abnormalities in the ExEn layer, characterized by discontinuously arranged, distended cells with sparse or absent microvilli. Consistent with the discontinuities in the ExEn of ZO-1^-/-^ ZO-2^-/-^ EBs, the ExEn was also abnormally permeable to macromolecules as large as IgG (150 kD). The increased permeability to a 40 kD fluorescent-dextran conjugate tracer was also reported for ZO-1 knockout/ZO-2 knockdown EpH4 epithelial cells [16]. The surface morphology and barrier abnormalities were also observed in ZO-1^-/-^ EBs, but to a much lesser degree, and they were restored to normal as in WT or ZO-2^-/-^ EB ExEn at later time points of culture. Interestingly, similar abnormalities have been reported in retinal pigment epithelium (RPE) deficient in ZO-1 protein [54]. The knockdown of ZO-1 caused the RPE to become riddled with gaps and disorganized, with flat and elongated cells lacking apical microvilli. While similar to the phenotype observed in ZO-1^-/-^ and ZO-1^-/-^ ZO-2^-/-^ ExEn, the RPE phenotype was associated with upregulation of epithelial-mesenchymal transition (EMT) markers. While we did not test for EMT markers, the outer cell layer in ZO-1^-/-^ and ZO-1^-/-^ ZO-2^-/-^ EBs differentiated into an epithelial layer of ExEn lineage based on the expression of ExEn differentiation markers such as Dab2 and Keratin-8.

The investigation of the underlying mechanistic pathways behind the aberrant ExEn morphogenesis led us to discover an anomalous expression of the transmembrane protein podocalyxin (PODXL), a positive regulator of microvilli and apical membrane domain [35] [34], at the ZO-1^-/-^ ZO-2^-/-^ ExEn. The normal continuous apical membrane expression of PODXL, as seen in WT and ZO-2^-/-^ EBs, was instead faint and fragmented in the ZO-1^-/-^ ZO-2^-/-^ ExEn. In ZO-1^-/-^ EBs, this partial abnormality recovered after prolonged culture. PODXL regulates apical membrane domain structure through its recruitment of the Ezrin-Radixin-Moesin (ERM) family member Ezrin [41] [42]. The deficient PODXL expression in ZO-1^-/-^ ZO-2^-/-^ ExEn correlated with an aberrant diffused submembranous localization of Ezrin, indicating that the PODXL-Ezrin pathway involved in apical membrane domain specification is deregulated in ZO-1^-/-^ ZO-2^-/-^ EB. Although probably not directly related to our context, the mislocalization of PODXL and activated Ezrin have been linked with the downregulation of Claudin-2 expression in epithelial cells [55]. In addition, PODXL-Ezrin play a role in organization of the apical actomyosin network [56] [41], a process in which ZO-1 and ZO-2 have also been implicated [57] [17].

Since PODXL expression at the ExEn apical membrane was abnormal, we speculated how this could be related to ZO-1 or ZO-2 protein function. The PODXL cytosolic tail has a C-terminal PDZ binding motif (DTHL) that is predicated to bind to Class I PDZ domains [58]. As the ZO proteins contain such PDZ domains [11], we tested the possibility of protein-protein interaction. The ZO proteins and PODXL did not co-localize or co-precipitate in WT EBs, indicating a lack of interaction under normal conditions (data not shown). Interestingly, quantitative real time-PCR analysis showed that the downregulation of PODXL expression in ZO-1^-/-^ ZO-2^-/-^ ExEn occurred at the transcriptional or mRNA stability level. In podocytes, the gene expression of PODXL can be suppressed by the combined effort of the transcription factor Wilms tumor 1 protein and PINCH1, an integrin signaling pathway adaptor protein [59]. Interestingly, PINCH1-null EBs partially phenocopy ZO-1^-/-^ ZO-2^-/-^ EBs, exhibiting loosely arranged, multilayered ExEn, unpolarized irregular arrangement of the primitive ectoderm (PrEc) cells and mislocalized basolateral or absent TJs [60]. Attempts to determine if the subcellular localization of PINCH1 was altered in ZO-1^-/-^ ZO-2^-/-^ ExEn were inconclusive (data not shown). In addition, a Wilms tumor 1-interacting protein has been shown to interact with ZO-1 and, during podocyte injury, co-localize with ZO-1 in the nucleus [61]. However, ZO-1 was not detected in the nucleus of ExEn cells of EBs.

Basement membrane deposition underlying the ExEn was fragmented and discontinuous in ZO-1^-/-^ ZO-2^-/-^ EBs compared with the continuous linear BM band in WT, ZO-1^-/-^ and ZO-2^-/-^ EBs. We speculate that this irregular deposition reflects the disorganized nature of the ZO-1^-/-^ ZO-2^-/-^ ExEn, which secretes the BM components [43]. This anomalous deposition in turn negatively affects the formation and survival of the PrEc epithelium, in agreement with the known relationship between the BM and PrEc morphogenesis [47] [48].

Related to the role of the BM on PrEc morphogenesis is the formation of large irregular cavities in the interior of ZO-1^-/-^ ZO-2^-/-^ EBs. These were not surrounded by discernible PrEc. ZO-1^-/-^ EBs displayed an intermediate phenotype with both normal cavities similar to WT and ZO-2^-/-^ EBs, as well as large cavities lacking a PrEc. We hypothesize that the lack of a survival signal emitted by an intact BM leds to the unregulated apoptosis of the EB interior, thus creating extensive cavities. Apoptotic bodies in EB cavities are normally removed by autophagy-regulated phagocytosis [62]. Surprisingly, in ZO-1^-/-^ ZO-2^-/-^ EBs, apoptotic cells progressively accumulated in the cavities, eventually filling up the lumen. This suggests a link between the absence of ZO-1^-/-^ ZO-2^-/-^ in EBs and autophagy or phagocytosis during cavitation. Alternatively, the deregulated cell death in ZO-1^-/-^ ZO-2^-/-^ EBs could be so extensive as to overwhelm the clearance of apoptotic cells. Interestingly, an accumulation of cell debris in the inter-retinal space has been reported when ZO-1 was depleted in RPE cells, suggesting the possible phagocytic dysfunction of these RPE cells [54].

In this study, we have explored the contributions of ZO-1 and ZO-2 to the normal morphogenesis of EB inner cell mass (ICM)-derived differentiated epithelium. ZO-2 null mutant EBs lacked an apparent phenotype for the processes analyzed, consistent with what has been reported in ZO-2 depleted immortalized mammalian epithelial cell-lines [63] [16]. In ZO-1 null mutants, we observed mutant phenotypes that were milder than those seen in the ZO-1 and ZO-2 double null mutants and which progressively recovered to resemble WT control EBs, suggesting that the lack of ZO-1 delays these processes during EB development, again consistent with data from MDCK cells where ZO-1 depletion delays TJ formation [63]. Considering that the combined deletions of ZO-1 and ZO-2 lead to more severe mutant phenotypes, we speculate that the two proteins function synergistically in EBs, with ZO-1 being more important in EB development.

The various mutant phenotypes of the ZO-1^-/-^ ZO-2^-/-^ EBs are mainly related to the abnormality of the ExEn, probably reflecting the absence of TJ formation. This EB outer layer is first derived from the ICM as the extraembryonic primitive endoderm, which subsequently differentiates into the visceral endoderm (VEn). The VEn plays critical roles in embryogenesis [64], thus the implications of its abnormality in ZO-1^-/-^ and ZO-1^-/-^ ZO-2^-/-^ EBs are significant. Importantly, the VEn is critical for development of the blood vessels in the yolk sac [65] and this may explain, at least in part, the deficiency in yolk sac angiogenesis reported in ZO-1 knockout mice [18]. Deletion of other genes involved in cell adhesion in mouse EBs phenocopy the ZO null EBs reported here. As mentioned earlier, PINCH1 null EBs partially phenocopy ZO-1^-/-^ ZO-2^-/-^ EBs. This is also the case for EBs lacking ILK, which links PINCH1 to the cytosolic tail of β-integrins at adhesion sites [66]. The mutant phenotypes of Afadin-deleted EBs also bear striking similarities to that of ZO-1^-/-^ and ZO-1^-/-^ ZO-2^-/-^ EBs. Afadin is an adherens junction (AJ) adaptor protein that can interact with ZO-1 and is important for the formation of TJs [67] [68]. Afadin-deleted EBs displayed impaired AJ and TJ formation, disorganized ExEn and PrEc, extensive unrestricted deposition of BM, and cell-filled cavities [69]. These similarities in EB mutant phenotypes of deleted genes involved in cell-cell or cell-substratum adhesion suggest a common underlying mechanism that will be explored in future work.

## Supporting Information

Figure S1 **A. PCR primers.** Nucleotide sequences of primers used for PCR and corresponding amplicon size in base pairs (bp) used in this study. **B, C**. **Antibodies**. List of primary (**B**) and secondary (**C**) antibodies used in this study.
(DOCX)

Figure S2 **Scanning electron micrographs.** Visualization of WT and knockout EB surface by SEM on culture Day-8 (**A**) and Day-10 (**B**). Panels b,d,f,h are magnifications of panels a,c,e,g respectively. WT (panels a and b) and ZO-2^-/-^ (panels e and f) EBs show regular cobblestone organization of ExEn epithelial cells at Day-8 and -10. The disorganized ExEn layer of ZO-1^-/-^ EBs at Day-5 (Fig. 3A, panel b) starts to become more regular (Fig. S1A, panel c, and panel d, bottom half) by Day-8, although some areas of disorganization persist (Fig. S1A, panel c, and panel d, top half). These disorganized areas are completely absent by Day-10 (Fig. S1B, panels c and d). Extensive disorder of ExEn cell arrangement is clearly seen in ZO-1^-/-^ ZO-2^-/-^ EBs (panels g and h) at Day-8 and -10, and does not recover to normalcy like in ZO-1^-/-^ EBs.
(EPS)

Figure S3 **Anti-PLC IgG-incubated permeability assay.** Panels b,d,f,h are magnifications of panels a,c,e,g respectively. Live EBs at Day-7 (**A**) and Day-10 (**B**) of culture were pre-incubated with anti-PLC IgG, which binds specifically to BM component Perlecan. This antibody was visualized with fluorophore-tagged secondary antibody (green color) after permeabilization of fixed EB sections. No anti-PLC IgG staining was observed in WT (panels a and b) and ZO-2^-/-^ (panels e and f) EBs at Day-7 and -10, indicating normal ExEn barrier function. Staining of the underlying BM was seen with ZO-1^-/-^ EBs at Day-7 (Fig. S2A, panels c and d) but this was absent at Day-10 (Fig. S2B, panels c and d). This implied that the ExEn permeability barrier was compromised earlier in ZO-1^-/-^ EB development but was restored to normalcy at later time points. Significantly, ZO-1^-/-^ ZO-2^-/-^ EBs (panels g and h) were stained extensively at both Day-7 and -10, implying severe compromise of the ExEn layer without any progressive recovery of barrier function. Nuclei are labeled with DAPI (blue color).
(EPS)

Figure S4 **Basement membrane immunostaining.** Fixed cryosections of Day-12 EB cultures were treated with antibodies immunoreactive to Perlecan (panels a-d), Collagen IV (panels e-h) and Laminin1+2 (panels i-l). This visualized the BM (red color, arrow) underlying the ExEn (arrowhead). Note that the BM of WT, ZO-1^-/-^ and ZO-2^-/-^ EBs formed as a continuous band, but the BM of ZO-1^-/-^ ZO-2^-/-^ EBs were fragmented and discontinuous. Nuclei are labeled with DAPI (blue color).
(EPS)
