# Supplementary material for: ZO-1 and ZO-2 Are Required for Extra-Embryonic Endoderm Integrity, Primitive Ectoderm Survival and Normal Cavitation in Embryoid Bodies Derived from Mouse Embryonic Stem Cells
Source: PLoS One. 2014 Jun 6;9(6):e99532. doi: 10.1371/journal.pone.0099532 (PMC4048262; doi:10.1371/journal.pone.0099532)
Supplement: Figure S1 — A. PCR primers. Nucleotide sequences of primers used for PCR and corresponding amplicon size in base pairs (bp) used in this study. B, C. Antibodies. List of primary (B) and secondary (C) antibodies used in this study. (DOCX) [file pone.0099532.s001.docx]

| **Gene** | **Forward** | **Reverse** | **Amplicon (bp)** |
| --- | --- | --- | --- |
| ZO-1 5’-Arm probe | GCA TGC TTG ACA CAG AGA AAT GGC | TGC AGG CCA AGC ACA CAA AAG GTA | 554 |
| ZO-2 5’-Arm probe | GAG TCT AGG AGG CTG TTC TTG TGT | ATG ACT CTT GAC CAG GGA GTC ACA | 566 |
| GATA4 | GGC GAG ATG GGA CGG GAC ACT | TGG GGC GCA TCT CTT CAC TG | 411 |
| GATA6 | ATC ACG GCG GCT TGG ACT GT | GTT CTC GGG GTT GGC GTT TTC | 364 |
| PODXL | TCG CCT GCA TCT CAC TCC CAT AAT | GCT CGC TGT GCT CGG TGA AGA AT | 504 |
| Ezrin | AGG TGG TAA AGA CGA TTG GCC TCC | GGT ATT CTA GCA TAG CAC TGT CCT | 480 |
| Pals1 | CAC AGT TAG AGC GGA TTC GAC AAC | TCA TGG GCC AGT AAA AGT GCC TGA | 514 |
| GAPDH | GCA AAT TCA ACG GCA CAG TCA A | GAG GGG CCA TCC ACA GTC TTC T | 415 |

**A**

**B**

| **Target Protein** | **Manufacturer** | **Cat. no.** | **Host** | **Application** |
| --- | --- | --- | --- | --- |
| ZO-1 | Life Technologies (Zymed) | 33-9100 | Mouse | IB/IF |
| ZO-2 | Cell Signaling Technology | 2847 | Rabbit | IB/IF |
| Claudin-6 | IBL-America | 18865 | Rabbit | IF |
| Laminin 1+2 | abcam | ab7463 | Rabbit | IF |
| Ezrin | abcam | ab41672 | Rabbit | IF |
| Collagen IV | abcam | ab6586 | Rabbit | IF |
| Troma-I | DSHB | - | Rat | IF |
| Dab2 | BD Transduction Laboratories | 610464 | Mouse | IF |
| PODXL | R&D Systems | AF1556 | Goat | IB/IF |
| Nidogen | Millipore (Chemicon) | MAB1946 | Rat | IF |
| Perlecan | Milipore (Chemicon) | MAB1948P | Rat | IF |
| Integrin α6 | Millipore (Chemicon) | MAB1378 | Rat | IF |
| GAPDH | Millipore (Chemicon) | MAB374 | Mouse | IB |

**C**

| **Target Ig** | **Manufacturer** | **Cat. no.** | **Host** |
| --- | --- | --- | --- |
| Anti-Mouse IgG-HRP | BIO-RAD | 170-6516 | Goat |
| Anti-Rabbit IgG-HRP | BIO-RAD | 170-6515 | Goat |
| Anti-Goat IgG-HRP | Santa Cruz Biotechnology | sc-2350 | Bovine |
| Anti-Mouse IgG-Alexa Fluor 488 | Life Technologies (Molecular Probes) | A-21202 | Donkey |
| Anti-Mouse IgG-Alexa Fluor 594 | Life Technologies (Molecular Probes) | A-21203 | Donkey |
| Anti-Rabbit IgG-Alexa Fluor 488 | Life Technologies (Molecular Probes) | A-21206 | Donkey |
| Anti-Rabbit IgG-Alexa Fluor 594 | Life Technologies (Molecular Probes) | A-21207 | Donkey |
| Anti-Rat IgG-Alexa Fluor 488 | Life Technologies (Molecular Probes) | A-21208 | Donkey |
| Anti-Rat IgG-Alexa Fluor 594 | Life Technologies (Molecular Probes) | A-21209 | Donkey |
| Anti-Goat IgG-Alexa Fluor 488 | Life Technologies (Molecular Probes) | A-11055 | Donkey |
